# Organic Hollow Mesoporous Silica as a Promising Sandalwood Essential Oil Carrier

**DOI:** 10.3390/molecules26092744

**Published:** 2021-05-07

**Authors:** Zuobing Xiao, Heqing Bao, Shuhan Jia, Yutian Bao, Yunwei Niu, Xingran Kou

**Affiliations:** School of Perfume and Aroma Technology, Shanghai Institute of Technology, Shanghai 201418, China; xzb@sit.edu.cn (Z.X.); 186071202@mail.sit.edu.cn (H.B.); 186071207@mail.sit.edu.cn (S.J.); 186071201@mail.sit.edu.cn (Y.B.); nyw@sit.edu.cn (Y.N.)

**Keywords:** sandalwood essential oil, organic hollow mesoporous silica, Pickering emulsion, loading capacity

## Abstract

As film-forming agents, fillers and adsorbents, microplastics are often added to daily personal care products. Because of their chemical stability, they remain in the environment for thousands of years, endangering the safety of the environment and human health. Therefore, it is urgent to find an environmentally friendly substitute for microplastics. Using *n*-octyltrimethoxysilane (OTMS) and tetraethoxysilane (TEOS) as silicon sources, a novel, environmentally friendly, organic hollow mesoporous silica system is designed with a high loading capacity and excellent adsorption characteristics in this work. In our methodology, sandalwood essential oil (SEO) was successfully loaded into the nanoparticle cavities, and was involved in the formation of Pickering emulsion as well, with a content of up to 40% (*w*/*w*). The developed system was a stable carrier for the dispersion of SEO in water. This system can not only overcome the shortcomings of poor water solubility and volatility of sandalwood essential oil, but also act as a microplastic substitute with broad prospects in the cosmetics and personal care industry, laying a foundation for the preparation and applications of high loading capacity microcapsules in aqueous media.

## 1. Introduction

In recent years, natural green additives used in daily chemical products have attracted immense interest. Natural plant essential oils, known as liquid gold, are a type of plant-derived secondary metabolites with small molecular weight steamed with water vapour. They have biodegradability and excellent antioxidant, antibacterial and other biological activities [1,2]. Therefore, they can find interesting applications in medicine, food and the daily chemical industry. Sandalwood (*Santalum album* L.), found in India, Malaysia, Australia and Indonesia, is considered to be one of the most valuable trees in the world. Sandalwood essential oil (SEO), obtained from the roots and trunk of sandalwood, is an industrial product widely used in medicine, chemicals and spices. SEO not only has bactericidal, antibacterial and antitumour functions, but also can relax nerves and relieve stress. Thus, it is widely used in cosmetics, perfumes and personal care products [3]. However, the application of SEO is greatly limited owing to its volatilisation, low water solubility and poor stability.

Nano-/microcapsule technology has the potential to overcome these problems. In the food industry, *Eucalyptus staigeriana* essential oil was loaded using cashew gum as wall material by Paula to increase its antibacterial activity [4]. The clove oil microcapsules were prepared by Wang [5]. Compared with clove oil, the microcapsules exhibited strong heat resistance and excellent antiseptic properties on meat products. Microcapsules with thyme oil as core material and maltodextrin as wall material were prepared by spray-drying [6]. The results indicated that the microcapsules had antibacterial effects on *Vibrio alginolyticus* and *Vibrio parahaemolyticus*. Nano-/microcapsule technology uses carriers to encapsulate unstable, reactive and volatile liquids or solids to form particles within a size range of 1 nm–1 mm. According to the type of materials, microcapsule carriers can be classified into polymer, inorganic and polymer-inorganic microcapsules [7]. Polymer microcapsules, such as polyethene and oxidised polyethene, are often added to personal care products, including toothpaste, detergents, facial cleansers and scrubs [8]. Nevertheless, due to their chemical stability, corrosion resistance and lack of biodegradation, they remain in the environment for hundreds and even thousands of years, seriously endangering the safety of the environment and life [9]. Consequently, there is an urgent need to find an environmentally friendly alternative to plastic microcapsules with a certain degree of friction resistance.

Silicon dioxide naturally exists in nature; the synthesis of mesoporous silica nanoparticles (MSNs) can be traced back to the early 1990s, and remarkable progress has been made since then [10,11]. Owing to their nontoxicity, high specific surface area, regular pore structure, adjustable pore size distribution, easy functionalisation, excellent mechanical stability and biocompatibility [12,13,14,15], MSNs have been widely used in spices [16], medicine [17], agriculture [18] and catalysts [19] as microencapsulated nanocarriers. On the contrary, their applications have been limited, due to their low loading capacity [20]. Hollow MSNs may be particularly valuable, due to their large internal space and pore structure, which can provide high loading capacity and slow-release characteristics for guest molecules [21,22,23,24].

Despite impressive achievements, the encapsulation of guest molecules in hollow silica materials is mainly achieved by the traditional impregnation method, which requires high hydrophilic and hydrophobic properties of encapsulated guest molecules. Due to the poor impregnation effect of hydrophobic guest materials, it is quite difficult for a large number of guest molecules to enter the cavity. Although the addition of cosolvents or phase transfer agents can enhance packaging efficiency, the introduction of supplementary additives can violate the principles of green chemistry.

Introducing hydrophobic groups on the surface of solid materials is beneficial to increase their hydrophobic properties, and thus, strengthen their adsorption to hydrophobic organic compounds in water [25,26,27]. Raspberry-like superhydrophobic hollow silica particles, which are modified by nonafluorohexyltriethoxysilane (NFH-silane) to reduce their surface energy, were obtained by Park and co-workers [28]. Unfortunately, although their adsorption properties can be increased, fluorine-containing modifiers are expensive and harmful to the environment [29]. Xu prepared a series of hydrophobic hollow silica nanospheres with *n*-octyltrimethoxysilane and other hydrophobic modifiers by the inverse emulsion method and selective etching [30]. However, stronger hydrophobicity of the hollow silica nanospheres was prone to result in a decreased dispersion in water, even lead to a float on water, which is a disadvantage for practical application. Moreover, stronger hydrophobicity does not always mean higher loading capacity, since the reduced dispersibility in water decreased their accessibility to the cargo compounds dissolved in water [31]. Therefore, adjusting the dispersion and distribution of hollow mesoporous silica in aqueous systems should not be neglected.

In this paper, novel hollow mesoporous silica with a high loading capacity and superior adsorption was designed using *n*-octyltrimethoxysilane (OTMS) and tetraethoxysilane (TEOS) as silicon sources. SEO was not only loaded in the nanoparticle cavities, but also revolved in Pickering emulsion as the dispersed phase. The effect of the amount of silicon source deposited on the shell, the additional amount of ammonium hydroxide solution, the proportion of OTMS to the silicon source and the additional amount of CTAC (cetyltrimethylammonium chloride) on the water contact angle, the dispersion of organic hollow mesoporous silica in water and the loading capacity of SEO were investigated, and organic hollow mesoporous silicas with high loading capacity and superior dispersion were prepared. The hollow mesoporous silica particles can substitute microplastics, making them attractive for applications in cosmetics and personal care products.

## 2. Results and Discussion

### 2.1. Experimental Preparation Process

**Scheme 1:**
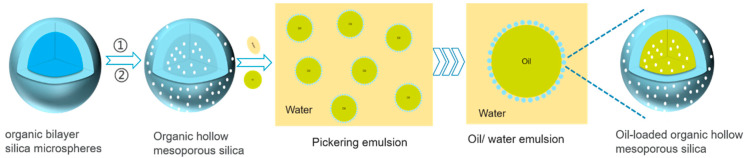
The preparation process of the whole experiment. **①** etched by sodium carbonate aqueous solution. **②** removing CTAC with acid and alcohol solution.

In this study, Scheme 1 illustrates the experimental methods. First, monodisperse silica nanospheres were synthesised by the hydrolysis of TEOS and the cross-linking of silicate. Then, the pore structure-directing agent CTAC was added to the solution. Considering that CTAC had both hydrophobic chains and hydrophilic groups, it can be arranged directionally to form micelles in the system, which can be radially adsorbed on the outer surface of negatively charged silica nanospheres by electrostatic adsorption. Consequently, the mixture of TEOS and OTMS was added, and the organic part of OTMS entered into CTAC micelles and enlarged the micelles [32]. Simultaneously, under the catalysis of the alkaline condition, the silicon source was hydrolysed and polymerised with positively charged CTAC. The change in charge density made the long chains of CTAC close to each other and further promoted the combination of the silicon source and the pore structure-directing agent. After condensation, organic bilayer silica microspheres were formed layer-by-layer on the surface of silica nanospheres. Furthermore, the organic bilayer silica microspheres were etched by a sodium carbonate aqueous solution at 80 °C. According to the research results of the Shi group [33,34], the degree of condensation between the inner cores of pure silica was lower than that of hybrid silica shells. Meanwhile, due to the protection of the hydrophobic layer on the surface of the hybrid shell, sodium carbonate selectively etched the core to form a hollow structure. CTAC played a key role in the formation of organic hollow mesoporous silica. However, it must be removed since it had a certain degree of cytotoxicity. The main methods of removing CTAC were extraction and calcination. But calcination required high temperatures (600 °C) for more than 4 h, causing the hollow structure to collapse. Therefore, reflux extraction with acid and alcohol was performed to remove CTAC and obtain organic hollow mesoporous silica connected by nanopores. SEO was added after the organic hollow mesoporous silica was uniformly dispersed in water. The adsorption capacity of SEO was enhanced, due to the hydrophobicity of the hybrid organic shell. After continuous stirring, SEO was adsorbed on the surface of the internal cavity of organic hollow mesoporous silica. This system formed a solution of oil in water, known as Pickering emulsion. Therefore, the loading capacity of organic hollow mesoporous silica greatly enhanced.

### 2.2. The Effects of Different Factors on the Water Contact Angle of Organic Hollow Mesoporous Silica and Its Loading Capacity to SEO 

The loading capacity of Pickering emulsion to SEO was used as an indicator for the single factor test. According to the results of the pre-experiment, the 3 mL of ammonium hydroxide solution, 30% (*v/v*) of OTMS to silicon source deposited on the shell and 335 mg of CTAC were selected for the following tests, respectively. 

#### 2.2.1. Effect of the Amount of Silicon Source Deposited on the Shell on the Water Contact Angle of Organic Hollow Mesoporous Silica and Its Loading Capacity to SEO

**Figure 1:**
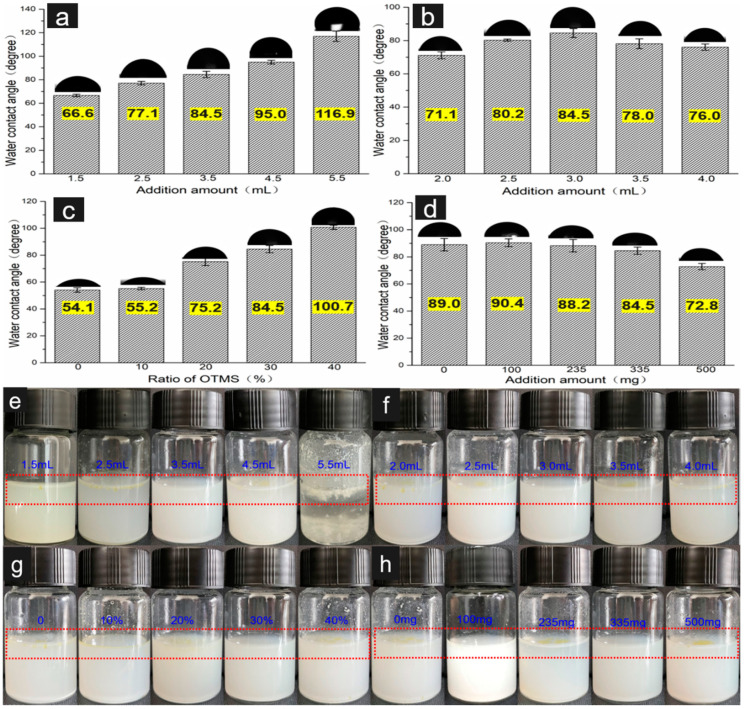
The effects of different factors on the water contact angle of organic hollow mesoporous silica and its loading capacity to SEO (**a**,**e**) The additional amount of silicon source deposited on the shell were 1.5 mL, 2.5 mL, 3.5 mL, 4.5 mL and 5.5 mL from left to right. (**b**,**f**) The ammonia hydroxide solution addition were 2.0 mL, 2.5 mL, 3 mL, 3.5 mL and 4 mL from left to right. (**c**,**g**) The ratio of OTMS to silicon sources deposited on the shell were 0, 10%, 20%, 30% and 40% (*v/v*) from left to right. (**d**,**h**) The additional amount of CTAC were 0, 100 mg, 235 mg, 335 mg and 500 mg from left to right.

**Figure 2:**
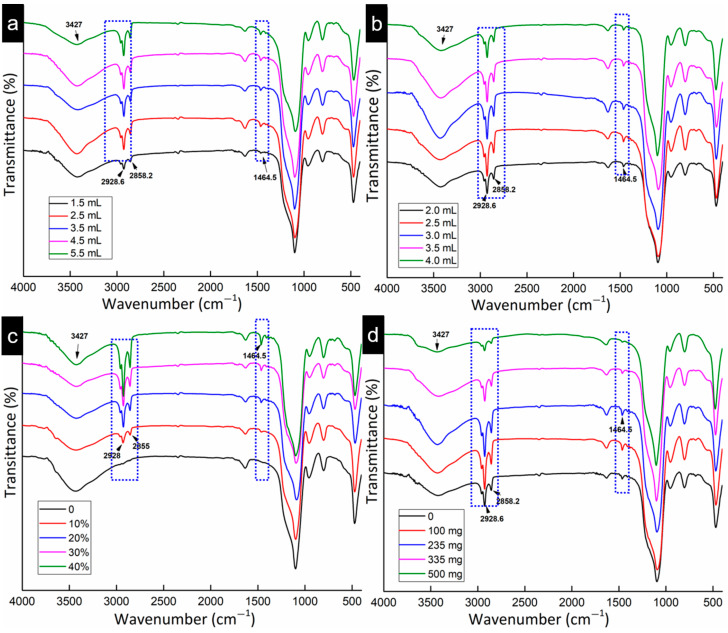
FTIR of organic hollow mesoporous silica formed by different influencing factors (**a**) The additional amount of silicon source deposited on the shell were 1.5 mL, 2.5 mL, 3.5 mL, 4.5 mL and 5.5 mL. (**b**) The ammonia hydroxide solution addition were 2.0 mL, 2.5 mL, 3 mL, 3.5 mL and 4 mL. (**c**) The ratio of OTMS to silicon sources deposited on the shell were 0, 10%, 20%, 30% and 40%. (**d**) The additional amount of CTAC were 0, 100 mg, 235 mg, 335 mg and 500 mg.

First of all, the effect of the amount of silicon source deposited on the shell on the water contact angle (WCA) of organic hollow mesoporous silica and its loading capacity to SEO was investigated. The addition amounts of silicon source deposited on the shell were 1.5 mL, 2.5 mL, 3.5 mL, 4.5 mL and 5.5 mL, respectively. The significant difference in WCA in Figure 1a reflected the difference in the wettability of solid materials obtained. With the increase in the amount of silicon source deposited on the shell, the WCA increased. The change of WCA may be caused by octyl groups [31]. In order to confirm our conjecture, FTIR was used to characterise the organic hollow mesoporous silica formed by the silicon source deposited on the shell with different addition amounts (Figure 2a). The absorption peaks around 2928.6 cm^−1^ and 2858.2 cm^−1^ were attributed to the antisymmetric and symmetrical stretching vibration of the functional group -CH_2_-. The changes at 1464.5 cm^−1^ may be attributed to the asymmetric angular vibration of CH_3_. -CH_2_- and CH_3_ were characteristic functional groups of OTMS. Their vibration amplitude gradually increased with the increase of the amount of silicon source deposited on the shell. When the mixed silicon source was added to the system, due to the hydrophobic silicon source possessing a nonpolar head, it was preferentially incorporated into the interior of CTAC. At this time, a small amount of TEOS was not involved in the reaction. With the increase in silicon sources, more octyl silicon sources were hybridised into the shells, and less TEOS entered into the shells. Therefore, the WCA increased with the increase of the amount of silicon source deposited on the shell.

Then 0.03 g of SEO was added to the aqueous solution of organic hollow mesoporous silica. The results showed that when the amount of silicon source deposited on the shell was 3.5 mL, the dispersion of the system was good, and no free-oil was observed (Figure 1e). This indicated that all the essential oils were embedded in organic hollow mesoporous silica. The SEO loading capacity was the highest when the addition of the silicon source deposited on the shell was 3.5 mL. The amount of SEO might be affected by the thickness of the organic hollow mesoporous silica shell. When the addition of silicon source was less than 3.5 mL, the spherical shell formed was so thin that it could not form a complete sphere, thus causing the structure to collapse. At the same time, due to its poor adsorption performance, the SEO could not actively enter the cavity, resulting in low loading capacity. Thus, it formed the free-oil. When the amount of silicon source deposited on the shell was greater than 3.5 mL, the WCA was greater than 90° (Figure 1a). However, because the spherical shell was too thick to enter the cavity, SEO was trapped in the mesoporous channel of the spherical shell. It was difficult to load a large amount of SEO. Therefore, the additional amount of silicon source deposited on the shell was selected as 3.5 mL to carry out the follow-up experiment.

#### 2.2.2. Effect of Ammonium Hydroxide Solution Addition on the WCA of Organic Hollow Mesoporous Silica and Its Loading Capacity to SEO

Secondly, the effect of ammonium hydroxide solution addition on the WCA of organic hollow mesoporous silica and its loading capacity to SEO was investigated. The addition amounts of ammonium hydroxide solution were 2 mL, 2.5 mL, 3 mL, 3.5 mL and 4 mL, respectively. As a catalyst, the amount of ammonium hydroxide solution had an important effect on the degree of the hydrolysis and condensation reaction of TEOS and OTMS. It can be seen from Figure 1b that the WCA of organic hollow mesoporous silica increased first and then decreased with an increase in the volume of ammonium hydroxide solution, which may be because ammonia as a catalyst had a great influence on the hydrolysis and condensation of organosilanes. It impacted the formation and accumulation rate of silane on the shell, thereby affecting the WCA. The FTIR (Figure 2b) was used to analyse the organic hollow mesoporous silica formed by different amounts of ammonium hydroxide solution. The vibration amplitude of the marked place increased first, and then decreased with an increase in the amount of ammonia addition, which was consistent with the results of the WCA, proving that the content of octyl functional groups in organic hollow mesoporous silica increased at first and then decreased. When the amount of ammonium hydroxide solution was less than 3.0 mL, the hydrolysis condensation rate increased with the increase in the ammonia volume, so the WCA increased gradually. When the volume of ammonium hydroxide solution exceeded 3.0 mL, the hydrolysis and condensation occurred not only at the oil–water interface, but also in the water phase, producing free silica particles and resulting in smaller WCA. 

Subsequently, 0.03 g of SEO was added to the aqueous solution of organic hollow mesoporous silica. Figure 1f clearly showed that when the amount of ammonia was 3 mL, no free-oil was observed. This meant that the loading capacity of SEO was the highest when the amount of ammonium hydroxide solution was 3.0 mL. The most likely reason was that when the amount of ammonium hydroxide solution was 3.0 mL, the WCA of the obtained solid material was the largest (Figure 1b). As a result of the strongest adsorption at this time, the loading capacity of SEO was the highest. Consequently, the additional amount of ammonia was selected as 3ml to carry out the follow-up experiment.

#### 2.2.3. Effect of the Ratio of OTMS to the Silicon Source Deposited on the Shell on the WCA of Organic Hollow Mesoporous Silica and Its Loading Capacity to SEO 

Thirdly, the effect of the ratio of OTMS to the silicon source deposited on the shell on the WCA of organic hollow mesoporous silica and its loading capacity to SEO was investigated. The proportions of OTMS to the silicon source deposited on the shell were 0, 10%, 20%, 30% and 40%, respectively. OTMS was a hydrophobic organosilicon source, which was hydrolysed and condensed with TEOS in the presence of ammonia to form a hybrid shell of organic hollow mesoporous silica. The ratio of OTMS to the silicon source had an important influence on the WCA of organic hollow mesoporous silica. In Figure 1c, the WCA of organic hollow mesoporous silica increased with the increase of the proportion of OTMS. The reason may be that OTMS was introduced into the surface of organic hollow mesoporous silica to form nanosised papillae, increasing the surface roughness of the material. At the same time, FTIR was used to characterise the organic hollow mesoporous silica formed by adding different OTMS ratios (Figure 2c). When OTMS was not added, no vibration was found at the 2928 cm^−1^, 2855 cm^−1^ and 1464 cm^−1^. With the increasing of OTMS, the amplitude of the vibration of the effective functional group became larger and larger. The fact that the formed organic hollow mesoporous silica contained more and more octyl groups was effectively confirmed. Therefore, the hydrophobic effect of the particles became stronger with an increase in the proportions of silicon sources deposited on the shell occupied by OTMS.

OTMS affected the WCA of organic hollow mesoporous silica, and thus, the loading capacity of SEO. Then 0.03 g SEO was added to the aqueous solution of organic hollow mesoporous silica. It was found that when the proportion of OTMS to the silicon source deposited on the shell was 30% (*v/v*), no free-oil was observed (Figure 1g). It may be that when the ratio of OTMS to silicon source deposited on the shell was less than 30% (*v/v*), the hydrophilicity of the formed organic hollow mesoporous silica was so high that the SEO cannot enter its internal cavities actively. When the proportion of OTMS to silicon source deposited on the shell was 40% (*v/v*), the WCA was 100.7° (Figure 1c). At this time, the surface energy of organic hollow mesoporous silica was too large to contact part of SEO dissolved in water. The loading capacity was relatively small, so some free-oil appeared. When the ratio of OTMS to silicon source deposited on the shell was 30% (*v/v*), the WCA was 85.4°. The surface energy and adsorption capacity were relatively good, so the loading capacity of SEO was larger. Therefore, the proportion of OTMS to the silicon source deposited on the shell was selected as 30% (*v/v*) to carry out the follow-up experiment.

#### 2.2.4. Effect of the Additional Amount of CTAC on the WCA of Organic Hollow Mesoporous Silica and Its Loading Capacity to SEO 

Fourth, the effect of the amount of CTAC on the WCA of organic hollow mesoporous silica and its loading capacity to SEO was investigated. The additions of CTAC were 0, 100 mg, 235 mg, 335 mg and 500 mg, respectively. Figure 1d shows the variation of the WCA of organic hollow mesoporous silica with the addition of CTAC. When the addition was less than 235 mg, the WCA of the organic hollow mesoporous silica mildly changed. The reason may be that CTAC as a surfactant affected the assembly of silicon sources. Due to the low concentration of CTAC at this time, the shell of the silicon source condensed into a saturated state after the addition of the silicon source. As a result, the WCA changed small. When the amount of CTAC continued to increase, the WCA of solid materials decreased. The reason may be that CTAC not only participated in the formation of pores, but also the excess CTAC and hydrophobic OTMS assembled to form polar head-outward hydrophilic molecules, resulting in the decrease of WCA. From the FTIR spectrum (Figure 2d), it can be seen that with the increase in the amount of CTAC, the vibration amplitude of effective functional groups increased at first and then decreased. This result was consistent with the results of WCA, indicating that the content of octyl functional groups in organic hollow mesoporous silica increased at first and then decreased.

**Figure 3:**
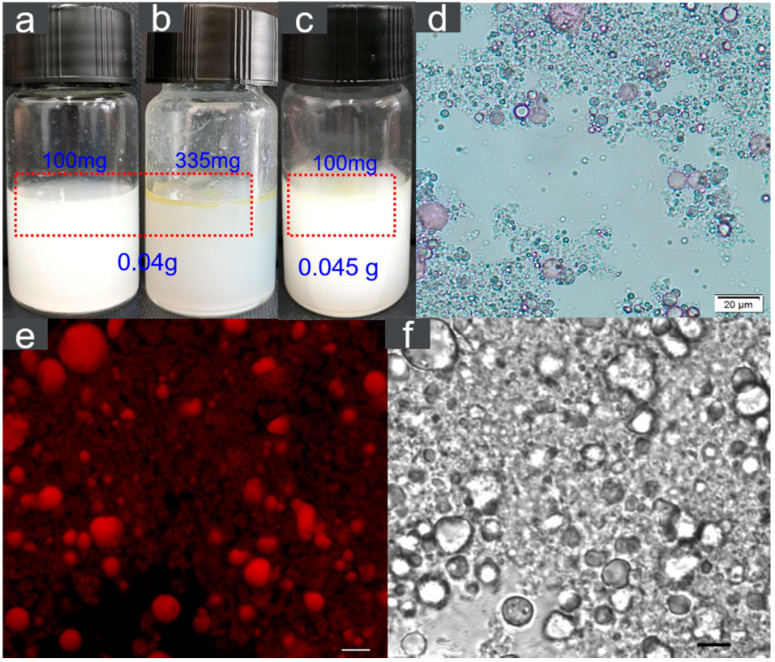
When the addition of SEO was 0.04 g, the oil-carrying performance of organic hollow mesoporous silica formed when the amounts of CTAC were 100 mg (**a**) and 335 mg (**b**), respectively. When the addition of SEO was 0.045, the oil-carrying performance of organic hollow mesoporous silica formed when the amount of CTAC was 100 mg (**c**). Organic hollow mesoporous silica Pickering emulsion with the highest oil loading was characterised by (**d**) microscope image, (**e**) CLSM dark field and (**f**) CLSM bright field, in which the scales of c and d were 10 μm.

CTAC impacted the pore formation of hollow mesoporous silica [35], and further affected its loading capacity. Subsequently, 0.03 g SEO was added to the aqueous solution of organic hollow mesoporous silica. The results showed that when the additional amount of CTAC were 100 mg and 335 mg, no free-oil was observed (Figure 1h). When the addition of SEO reached 0.04 g, free-oil appeared in the sample with CTAC addition of 335 mg (Figure 3b). Therefore, when the addition of CTAC was 100 mg, the prepared organic hollow mesoporous silica had the best loading capacity and dispersion. In order to measure the loading capacity, the addition of SEO was further increased to 0.045 g. It was found that once 0.045 g of SEO was added, free-oil appeared in the sample (Figure 3c). According to the calculation formula of the loading capacity:

$$\mathrm{LC}\;\left(\%\right)=\frac{w_{1}}{w_{1}{+w}_{2}}\times \;100\%$$

Among them, LC (%) is loading capacity of organic hollow mesoporous silica to SEO, w_1_ is maximum addition amount of SEO when free-oil did not appear, w_2_ is addition amount of organic hollow mesoporous silica.

Thus, the loading capacity of organic hollow mesoporous silica to SEO was 40% (*w/w*). Compared with non-biodegradable materials, such as polyacrylamide and polymethyl methacrylate, the load may not be large enough, but it has a bright future and great potential [36]. Notably, the hollow mesoporous silica formed by 100-mg CTAC had the highest loading capacity of SEO, which may be because CTAC micelles affected the formation of mesoporous silica. With the increasing addition of CTAC, the mesopores became smaller, and their loading capacity decreased. When the WCA was 90.4° (Figure 1d), SEO was not only adsorbed into the internal cavity of organic hollow mesoporous silica, but also formed Pickering emulsion with organic hollow mesoporous silica. In order to confirm our conjecture, the emulsion was observed by an Olympus microscope and confocal laser scanning microscope. As shown in Figure 3d, obvious milk droplets can be seen without adding any surfactant. Meanwhile, under the laser confocal microscope (Figure 3e,f), it was observed that SEO labelled with Nile red and organic hollow mesoporous silica formed oil in water (O/W) Pickering emulsion. Therefore, the SEO loading capacity of organic hollow mesoporous silica was the highest. 

In short, through the above single factor experiment, it was concluded that when the 3.5 mL of silicon source deposited on the shell, 3 mL of ammonium hydroxide solution, 30% (*v/v*) of OTMS to silicon source deposited on the shell and 100 mg of CTAC were selected, respectively, the prepared organic hollow mesoporous silica had the highest loading capacity (40% (*w/w*)).

### 2.3. Micro-Morphological Characterisation of Organic Hollow Mesoporous Silica

**Figure 4:**
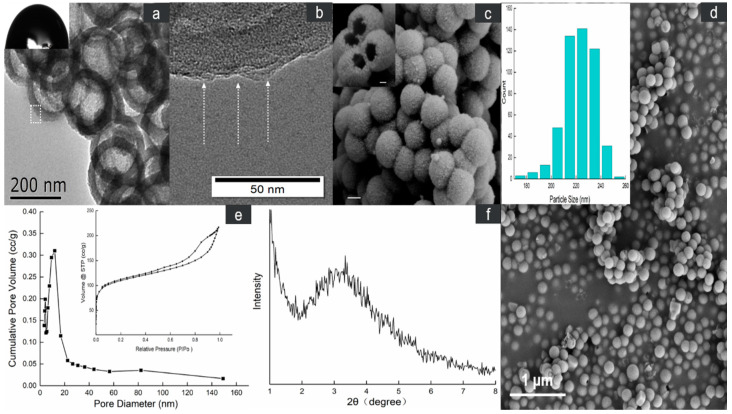
(**a**,**b**) TEM,(**c**,**d**) SEM, (**e**) pore size distribution curve under desorption pattern, and (**f**) SMXRD images of organic hollow mesoporous silica with the highest oil-loading r. Insert in (**a**) shows WCA image. The scale in (**c**) was 100 nm. Insert in (**c**) shows a partially crushed sphere, in which the scale was 50nm. Insert in (**d**) shows the average particle size. Insert in (**e**) shows the isotherm image.

Figure 4a shows the TEM image of hollow mesoporous silica with the highest oil-loading. The inner particle diameter was about 175 nm. The water contact angle was 90.4° (Figure 4a insert). The high-magnification TEM image (Figure 4b) revealed that the shell of the hollow mesoporous silica displayed an obvious mesoporous structure. The hollow structure was formed by the etching of a sodium carbonate aqueous solution. It was worth noting that the ground particles (Figure 4c insert) clearly showed a hollow structure, which was quite consistent with the TEM observations. The microspheres in the SEM image (Figure 4d) were measured through the particle size statistics software (Nano Measurer 1.2) to obtain the average particle size of the organic hollow mesoporous silica (Figure 4d insert). The average particle size was 222 nm, which was consistent with the results of TEM and SEM. The pore size distribution curve and adsorption isotherm image were obtained by Brunner-Emmet-Teller (BET), as shown in Figure 4e. The total volume of pores was 3.354 cm^3^ g^−1^, which was determined with density functional (DFT) methods. According to Barret-Joyner-Halenda (BJH) desorption summary, the surface area and pore volume of mesopores were 86.48 m^2^ g^−1^ and 0.188 cm^3^ g^−1^, respectively. The micropore width, micropore volume and micropore surface area of the hollow mesoporous silica were 1.45 nm, 0.118 cm^3^ g^−1^ and 284.6 m^2^ g^−1^, respectively, which were determined with the Langmuir method. According to the classification of IUPAC, the nitrogen adsorption isotherm showed an IV-type curve, and there was an H3-type hysteresis curve in the P/P_0_ range, which was steeper than the adsorption branch. The results showed that the shell had a typical hollow structure with nano-mesoporous channel connectivity. The (BJH) pore size distribution curve showed two peaks. The first peak corresponds to the mesoporous structure with an average pore size of 3.3 nm. The pore size distribution of the peak indicated the mesoporous structure. The last peak also indicated a mesopore distribution near 10 nm, which may be attributed to the hollow space of organic hollow silica. This further proved the coexistence of the mesoporous structure and hollow structure of organosilicon [37,38]. The XRD pattern (Figure 4f) showed a diffraction peak around 2θ = 3°, which was similar to the results of the reported mesoporous silica structure [37,39], further proving the mesostructure in the shells. The result was consistent with the channel distribution curve observed in Figure 4e (inset diagram) and Figure 4b.

**Figure 5:**
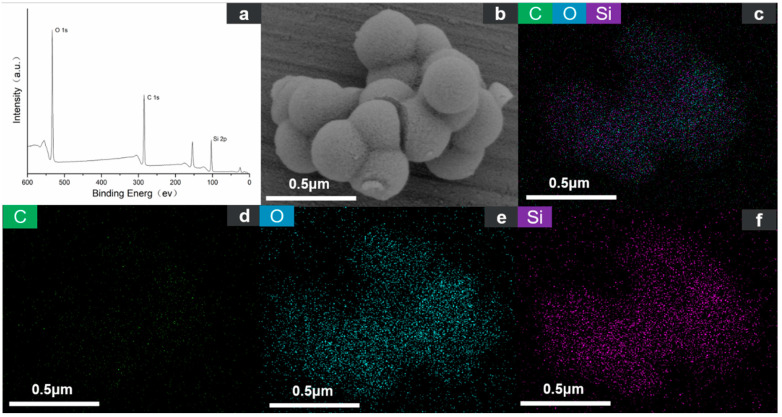
(**a**) XPS, and (**b**–**f**) EDX element distribution images of organic hollow mesoporous silica with the highest oil-loading and dispersion in water. Green, blue and purple denoted C, O and Si, respectively. The scales in (**b**–**f**) was 0.5 μm.

To explore the elemental composition on the surface of organic hollow mesoporous silica, it was tested by XPS and EDS. As shown in Figure 5a, a group of peaks for Si 2p, C1s and O1s can be observed near 103 eV, 284 eV and 532 eV, respectively. Energy-dispersive X-ray (EDX) analysis (Figure 5b–f) showed that carbon, oxygen and silicon were located on the shell. XPS and EDX elemental distribution images showed that C, O and Si appeared on organic hollow mesoporous silica, indicating that OTMS and TEOS were hydrolysed and condensed successfully to form the silicon shell.

### 2.4. FTIR Analysis of Organic Hollow Mesoporous Silica and Its SEO Microcapsules

**Figure 6:**
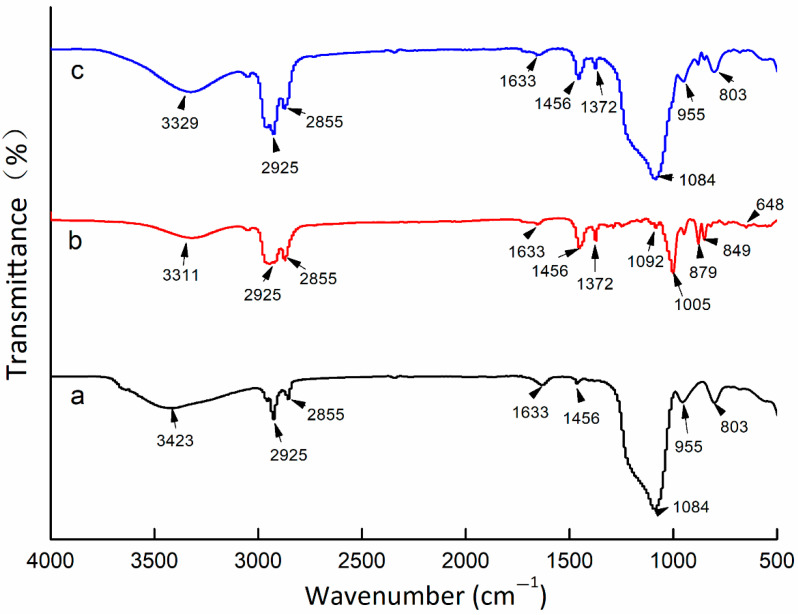
FTIR of organic hollow mesoporous silica blank wall material (**a**), SEO (**b**) and organic hollow mesoporous silica SEO microcapsule (**c**).

The organic hollow mesoporous silica with the highest oil-loading and dispersion in water was prepared. Then, organic hollow mesoporous silica Pickering emulsion was further prepared using this material. The organic hollow mesoporous silica SEO capsules were obtained by the freeze-drying method. The molecular structure and intermolecular force of SEO, organic hollow mesoporous silica and its SEO microcapsule were characterised by FTIR. As shown in Figure 6 and Table 1, the main characteristic peaks were marked in the spectrum.

In spectrum 6 (a), there was a typical broad absorption peak corresponding to the stretching vibration of Si-OH near 3423 cm^−1^, while the absorption peaks around 2925 cm^−1^ and 2855 cm^−1^ were attributed to the antisymmetric and symmetrical stretching vibration of the functional group -CH_2_- in OTMS, indicating the existence of organic group -CH_2_- in organic hollow mesoporous silica. The absorption peak at 1456 cm^−1^ was caused by the bending vibration of the CH_3_ group. The absorption peaks near 1084 cm^−1^ and 803 cm^−1^ were attributed to the stretching vibrations of the -Si-O-Si- group and Si-O group [40], while -Si-O-Si- and Si-O were the characteristic functional groups of organic silicon compounds. The appearance of absorption peaks of 2925 cm^−1^, 2855 cm^−1^, 1456 cm^−1^ and 1084 cm^−1^, 803 cm^−1^ indicated that the hydrolysis and condensation reaction between OTMS and TEOS was performed, and organic hollow mesoporous silica was successfully formed.

Compared with the spectra in (a–c) in Figure 6, it was obvious to observe that the broad absorption peak of the O-H group near 3423 cm^−1^ shifted to low frequency, and the vibration amplitude increased, which was due to the stronger intermolecular or intramolecular hydrogen bonding formed between O-H groups in the compound molecule. Although the three spectra showed obvious absorption peaks near 2925 cm^−1^ and 2855 cm^−1^, the strength in spectra c was higher than that in a and b, which indicated that SEO was successfully adsorbed in the cavity of organic hollow mesoporous silica, and the vibration amplitude was increased. In the spectra of a, b and c, a typical absorption band at 1633 cm^−1^ was assigned to the bending vibration of H_2_O [27]. The changes at 1456 cm^−1^ and 1372 cm^−1^ in Figure 6b,c may be attributed to the asymmetric angular vibration and symmetrical angular vibration of CH_3_, which might be caused by the destruction of the state of the Pickering emulsion during drying, resulting in the exposure of some SEO to the outside of organic hollow mesoporous silica. In spectrum 6 (b), the stretching vibrations of the C-OH group in the alcohol molecule, which were near 1092 cm^−1^, 1005 cm^−1^ and 648 cm^−1^, all disappeared in spectrum c. Meanwhile, the bending vibration absorptions of the C-H group, which were near 879 cm^−1^ and 849 cm^−1^, were weakened. The changes in the characteristic peaks above showed that SEO was successfully adsorbed in the cavity of organic hollow mesoporous silica.

### 2.5. Thermal Stability of SEO, Organic Hollow Mesoporous Silica and Organic Hollow Mesoporous Silica SEO Microcapsule

**Figure 7:**
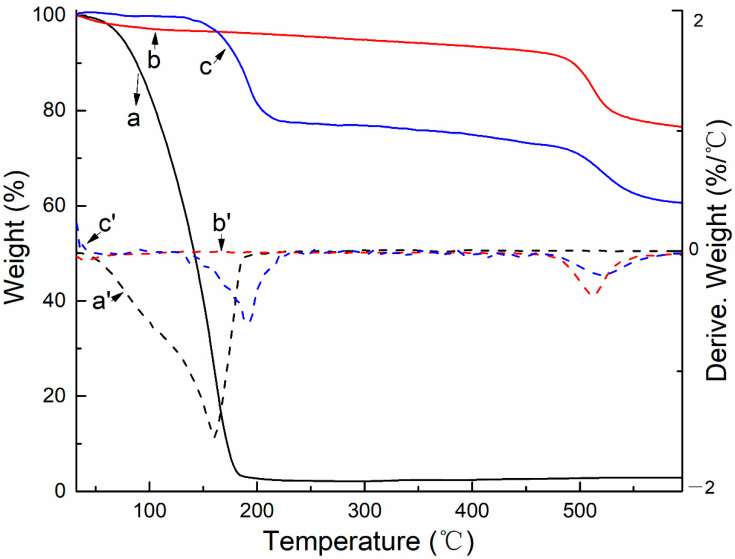
TG and DTG curves of SEO (**a**,**a’**), organic hollow mesoporous silica (**b**,**b’**) and organic hollow mesoporous silica SEO microcapsule (**c**,**c’**), respectively.

The TG curve and DTG curve of SEO (a,a’), organic hollow mesoporous silicon (b,b’) and organic hollow mesoporous silica SEO microcapsule (c,c’) had been shown in Figure 7. As can be seen from curve a, the quality of SEO gradually disappeared with the increase of temperature. Most of the mass loss was in the range of 50–186 °C. The maximum thermal decomposition rate occurred at 160 °C, as shown in curve a’. The mass loss of curves b and c also occurred at 65–70 °C, which may be caused by the volatilisation of water molecules in the organic hollow mesoporous silica cavity. The mass loss of curve b was about 22% at 130–230 °C. It can be seen from Figure 7b’ that the mass rate of this stage decreased quickly. The boiling point of sandalwood alcohol, the main component of SEO, was about 168 °C. The thermal instability of SEO made it released from the cavity of organic hollow mesoporous silica. However, due to the protection of organic hollow mesoporous silica, the volatilisation rate of SEO reached the maximum at 191 °C. Compared with curve a, the thermal stability of SEO wrapped by organic hollow mesoporous silica was obviously improved. The losses of curve b and curve c at 470–545 °C were 23.8% and 17%, respectively. The mass loss at this stage may be due to the decomposition of carbon in organic hollow mesoporous silica. This showed that the thermal stability of organic hollow mesoporous silica was excellent. In summary, through the TG and DTG patterns, it can be seen that the initial volatilisation temperature of SEO was 50 °C, and the volatilisation rate reached the maximum at 160 °C, and almost evaporated at 186 °C. On the other hand, the SEO encapsulated by silica began to volatilise at 130 °C, the fastest volatilisation rate was at 191 °C, and then evaporated at 230 °C. Therefore, SEO was encapsulated by organic hollow mesoporous silica, which can not only reduce the volatility of SEO, but also improve the thermal stability of SEO.

### 2.6. Stability of the Pickering Emulsion with Various Organic Hollow Mesoporous Silica Concentrations

**Figure 8:**
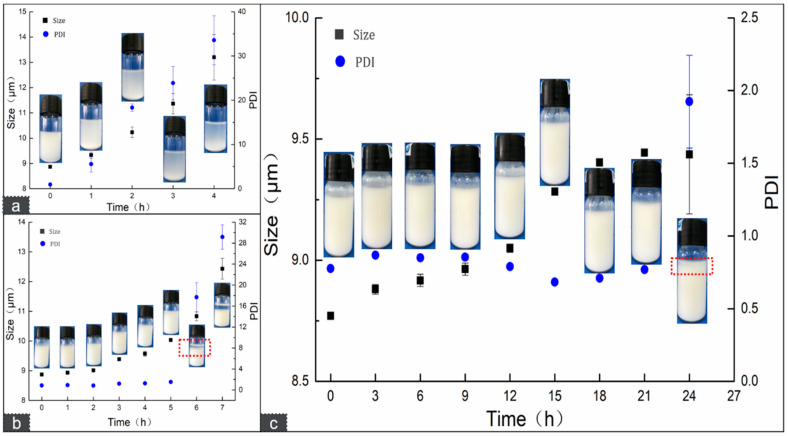
Variation of particle size and PDI of Pickering emulsion of organic mesoporous silica with different concentrations over time. The concentrations in Figure (**a**–**c**) were 1%, 10% and 20%, respectively.

Figure 8 showed the variation of particle size and PDI of Pickering emulsion of organic mesoporous silica with different concentrations over time. With the increase of the concentration of organic hollow mesoporous silica, the stability of Pickering emulsion increased from 0 h (Figure 8a) to 21 h (Figure 8c), while the particle size decreased from 8.95 μm to 8.78 μm. This may be due to the increase of particle concentration in the system—enough particles were adsorbed at the interface and form a dense interface film, which improved the surface coverage and led to the formation of network structure around the emulsion droplets. This structure formed the skeleton of the emulsion and inhibited the coalescence of droplets, further improving the stability of the emulsion [41,42]. From Figure 8c, it was observed that the particle size increased with time. PDI was relatively stable within 21 h, but increased suddenly at 24 h. The photos (Figure 8c) of the emulsion also showed that delamination began to occur at 24 h. These results showed that the organic hollow mesoporous silica Pickering emulsion can be stabilised for 21 h at room temperature. 

## 3. Materials and Methods

### 3.1. Chemicals and Materials

Cetyltrimethylammonium chloride (99%, CTAC), tetraethoxysilane (99%, TEOS) and n-octyltrimethoxysilane (97%+, OTMS) were kindly provided by Adamas-beta, Shanghai Titan Scientific Co., Ltd. Ammonium hydroxide solution (AR, 25–28%) and ethanol anhydrous (≥99.7%, AR) were purchased from General-reagent, Shanghai Titan Scientific Co., Ltd (Shanghai, China). Sodium carbonate anhydrous (AR) and hydrochloric acid (AR, HCl) were from Sinopharm Chemical Reagent Co., Ltd (Shanghai, China). Nile Red (99%, pure) and SEO were obtained from Shanghai Titan Scientific Co., Ltd. Deionized water made by Shanghai Institute of Technology was used in all experiments. 

### 3.2. Preparation of Organic Hollow Mesoporous Silica

According to the improved Stöber method [43], 10 mL deionised water, 70 mL ethanol anhydrous and a certain amount of ammonium hydroxide solution were successively poured into a conical bottle with a magnetic agitator, and the mixture was stirred at 600 rpm for about 30 min at 35 °C. The initial pH of the reaction was 11. After this, 6 mL of TEOS was added to the mixture. Subsequently, the reaction solution was stirred for 1 h to form a milky silica colloidal suspension. Following, a certain amount of CTAC was added. After stirring for 30 min, a certain proportion of the mixture of OTMS and TEOS used as silicon source was added. The hydrolysis and condensation of TEOS and OTMS reacted for 1.5 h. At last, through centrifugation and washing (three times), the white powders of organic bilayer silica microspheres were successfully prepared. Then 3.75 g of the obtained product was dispersed in 100 mL 21.2 mg/mL sodium carbonate aqueous solution and stirred at 80 °C for 50 min to convert the products into organic hollow silica microspheres. After centrifugation and washing, they were mixed into 200 mL ethanol solution containing 1.6 mL concentrated hydrochloric acid and refluxed at 125 °C for 3 h to remove CTAC. Eventually, through centrifugation, washing and vacuum drying, the final product of organic hollow mesoporous silica was obtained.

### 3.3. Preparation of a Solution System of Organic Hollow Mesoporous Silica and SEO

0.06 g organic hollow mesoporous silica powder was ultrasonically dispersed in 9.9 mL deionised water, and SEO was added. The emulsions of organic hollow mesoporous silica and SEO were obtained by magnetic stirring with 500 rpm/min at 25 °C and mixed by vortexing for 3 h.

### 3.4. Stability of the Pickering Emulsion with Various Organic Hollow Mesoporous Silica Concentrations

Although the loading capacity and dispersion of the emulsion obtained from the single factor test are the best, its stability is not so satisfactory. It is reported that the stability of the emulsion is closely related to the wettability, size, shape, surface properties and particle concentration of the particles [42]. In order to get a more stable emulsion, the concentration of the particles was changed. 0.06 g hollow mesoporous silica powder was uniformly dispersed in 9.9 mL deionised water. Then 0.04 g SEO was added. The Organic hollow mesoporous silica with a concentration of 1% was obtained by magnetic stirring with 500 rpm/min at 25 °C and mixed by vortexing for 3 h. Using the same method, organic hollow mesoporous silica Pickering emulsions with concentrations of 10% and 20% were prepared, respectively.

### 3.5. Characterisation of Micro-Morphology and Physicochemical Properties of Organic Hollow Mesoporous Silica

Transmission Electron Microscopy (TEM) images were acquired using a jeol2100f TEM with a field emission gun operated at 200 kV. Microparticle morphology was observed by field-emission SEM (Sigma 300, Oberkochen, Germany). The dried powder samples were fixed on a metal stub with the aid of double-sided tape, then sputter-coated with gold. Finally, it was detected by Inlens and ET secondary electron detectors and Smart EDX spectrometer, and the images were obtained on a field-emission SEM at 15.0 kV. N_2_ physical adsorption was performed on accelerated surface area and porosity analyser (ASAP 2460) under a continuous adsorption condition. Pore diameters were calculated from the desorption branch of the isotherm with the BJH method. Small-angle X-ray diffraction (SAXRD) patterns of the prepared materials were noted down with a Japanese Neo-Rigaku Ultimate IV diffractometer. The model K-Alpha (Thermo Fisher) X-ray photoelectron spectrometer (XPS) was used to detect the organic hollow mesoporous silica and determine the elemental composition of its surface. The water contact angle (WCA) was measured by Theta flex optical contact angle meter (Biolin scientific AB, Gothenburg, Sweden). The droplet volume is 5 μL, and the angle accuracy is ±0.5°. Before the test, the organic hollow mesoporous silica was pressed into a uniform sheet by a pressing machine (FW-4, Tianjin skylight new optical instrument technology CO., LTD, Tianjin, China) under the pressure of 20 MPa, and then tested. 

### 3.6. Characterisation of Morphology and Properties of Organic Hollow Mesoporous Silica Pickering Emulsion

0.04 g of SEO marked with Nile red was mixed with 9.9 mL water and 0.06 g uniform solution of organic hollow mesoporous silica. After mixing by vortexing for 3 h, the morphology of the emulsion was observed by Olympus microscope (model: BX53, Shanghai, China) and confocal laser scanning microscope (CLSM, model: FV1200, Shanghai, China). The Fourier transform infrared (FTIR) analysis was carried out on Thermo Scientific Nicolet iS5 infrared spectrometer. Malvern laser particle size analyser Mastersizer 3000 and static observation method were used to characterise the stability of the Organic Hollow mesoporous silica Pickering Emulsion. Thermal stability results were tested by a thermogravimetric (TG) analyser (Netzsch STA 449F3, Selb, Germany).

## 4. Conclusions

In this study, organic hollow mesoporous silica was prepared using OTMS as an organic hydrophobic agent. The effects of the amount of silicon source deposited on the shell, the addition of ammonium hydroxide solution, the proportion of OTMS and the addition of CTAC on the WCA of organic hollow mesoporous silica and its loading capacity to SEO were investigated. We found that organic hollow mesoporous silica Pickering emulsion can be relatively stable at room temperature for 21 h without using any surfactant, and the loading capacity can be up to 40% (*w/w*). Sandalwood essential oil (SEO), which is hydrophobic, was successfully dispersed in the aqueous phase, overcoming the shortcomings of poor water solubility. At the same time, the TG images showed that the organic hollow mesoporous silica improved the volatility and thermal stability of SEO. SEO can not only be encapsulated in the cavity of organic hollow mesoporous silica microspheres, but also can form a Pickering emulsion with organic hollow mesoporous silica microspheres, which further increases the loading capacity of SEO. This system can act as a microplastic substitute with broad prospects in the cosmetics and personal care industry, laying a foundation for the preparation and applications of high loading capacity microcapsules in aqueous media. In addition, we will continue to improve the system to prolong its stability so that the aroma in daily chemical products can be released in multiple stages and remain fragrant for an extended time.

## Figures and Tables

**Table 1:** Vibrational band shifts and their respective assignments in Fourier transform infrared spectroscopy.

Wavenumber (cm^−1^)			The Reason for the Change of Wavenumber
a	b	c	
3423	3311	3329	the stretching vibration of O-H
2925	2925	2925	the antisymmetric and symmetrical stretching vibration of the functional group -CH_2_-
2855	2855	2855	
1633	1633	1633	the bending vibration of H_2_O
1456	1456	1456	the asymmetric angular vibration and symmetrical angular vibration of CH_3_
	1372	1372	
	1092		the stretching vibrations of the C-OH group in the alcohol molecule
1084		1084	the stretching vibrations of -Si-O-Si- group
	1005		the stretching vibrations of the C-OH group in the alcohol molecule
955		955	the stretching vibrations of -Si-O-Si- group
	879,849		the bending vibration absorption of the C-H group
803		803	the stretching vibrations of -Si-O- group
	648		the stretching vibrations of the C-OH group in the alcohol molecule

## Data Availability

Not applicable to this article.

## Sample Availability

Samples of the compounds are available from the authors.
